# Anterior Cruciate Ligament Injuries: A Review of Quality and Reliability of Online Information

**DOI:** 10.7759/cureus.75776

**Published:** 2024-12-16

**Authors:** Ahmad W Mohamed, Matthieu Durand-Hill, Arpit Patel

**Affiliations:** 1 Trauma and Orthopaedic Surgery, Addenbrooke's Hospital, Cambridge University Hospitals NHS Foundation Trust, Cambridge, GBR; 2 Trauma and Orthopaedics, London North West University Healthcare NHS Trust, London, GBR; 3 Trauma and Orthopaedics, Broomfield Hospital, Mid and South Essex NHS Foundations Trust, Chelmsford, GBR

**Keywords:** acl, acl injury, acl sprain, anterior cruciate ligament (acl) reconstruction, ortho, ortho surgery, sports surgery

## Abstract

Aim

This study aims to evaluate the reliability and quality of online information on anterior cruciate ligament (ACL) injuries.

Methods

An internet search on the three top search engines, Google, Yahoo!, and Bing, was done using the keywords “anterior cruciate ligament injury”. The search was carried out in June 2023, and 39 websites were selected. Exclusion criteria comprised video-only explanatory websites (such as YouTube) and access requiring payment or registration. Websites were categorised using the following scoring systems: (i) DISCERN score, (ii) Health-on-Net Foundation Code (HON code), (iii) Journal of the American Medical Association (JAMA) benchmark criteria, and (iv) ACL content score, which was specifically designed for this study.

Results

The majority of websites were commercial (n = 16 [41.0%]), followed by academic (n = 10 [25.6%]). None of the websites included had a HON code present. The mean DISCERN score was 52.1, the mean JAMA score was 2.62, and the mean ACL content score was 7.49.

Conclusion

There is a vast amount of information available on the internet with regard to the topic of ACL injuries, and this ranges from excellent to poor-quality information. In light of this, orthopaedic specialists and healthcare providers must guide patients to online resources that are reliable and trusted based on either their personal experience or resource type (academic/physician). In addition, we recommend that websites providing information seek HON-code certification as a seal of quality, as this has previously been noted in previous studies to be positively linked with the quality of information delivered.

## Introduction

Anterior cruciate ligament (ACL) injuries are common, specifically in the field of sports injuries, with a reported incidence of 100,000 to 200,000 in the United States every year [1]. Age-specific patterns differ in male and female patients, with a peak in incidence between 19 and 25 years in males and a peak in incidence between 14 and 18 years in females [2]. Given the age group and demographic of these patients, there is a propensity to seek out information about their injury from various online resources, either prior to visiting specialist clinicians or for further reading following consultations [3]. This tendency can have both positive and negative effects on the patients, depending on the quality and accuracy of the information they read. Some studies demonstrated that poor-quality information negatively impacted patients [4]. The information sought will vary in understanding the injury itself, first aid needed, treatment options, and sequelae of the injury. There are plenty of online resources providing information on this injury; however, the quality and reliability of them are unclear. Literature shows that patients who have a better understanding of their injury are more likely to have better outcomes [5]. Furthermore, studies have demonstrated that patient education can reduce healthcare costs by reducing the frequency of visits to healthcare providers [6]. The purpose of this study is to assess the quality and reliability of the information provided online on ACL injuries.

## Materials and methods

An internet search on the three top search engines [7], Google, Yahoo!, and Bing, was done using the keywords “anterior cruciate ligament injury”. The search was carried out in June 2023, and 39 websites were selected. Exclusion criteria comprised the following: Video-only explanatory websites (such as YouTube), websites requiring paid access, websites requiring membership registration, and finally online books (eBooks).

Websites were assessed by two senior orthopaedic residents. They were categorised as follows: (i) government-produced, (ii) academic, (iii) commercial, (iv) produced by physicians, and (v) produced by allied health professionals. Then resources were assessed using the following scoring systems: (i) DISCERN score, (ii) Health-On-Net Foundation Code (HON code), (iii) Journal of the American Medical Association (JAMA) benchmark criteria, and (iv) ACL content score, which was specifically designed for this study. When a website's evaluation against a specific criterion was uncertain, the assessors engaged in a discussion to reach a final consensus.

DISCERN is an instrument or tool that has been designed to help users of consumer health information judge the quality of written information about treatment choices [8]. It has 16-point questions, which sum up to a final score, the maximum being 80. Each question is rated from 1-5. Questions 1-8 address the reliability of the publication and should help you consider whether it can be trusted as a source of information about treatment choices. Questions 9 - 15 focus on specific details of the information about treatment choices. Question 16 is the overall quality rating at the end of the instrument [8].

The Health-on-the-Net Foundation was a non-profit organisation with the aim of identifying and reporting reliable, trustworthy, and comprehensive sources of online health information [9]. Websites providing information that complies with their eight-point code of conduct are allowed to display the seal but are subject to random verification checks for compliance. The authors are aware that it has been discontinued since 2022, but most websites included in the review had been established prior to that, and it was therefore included in this paper.

The JAMA benchmark criteria tool was initially published in a paper by Silber et al. in 1997. It is made up of four core standards: authorship, attribution, disclosure, and currency. A point for each standard is given for a clear presentation of each [10].

The ACL content score was designed for this study following review by orthopaedic surgeons specialised in soft tissue knee injuries. It comprises 11 aspects relating to the injury. A point is given for a clear demonstration of each, giving a maximum score of 11. The 11 points are: (i) anatomy and function of the ACL, (ii) mechanism(s) of injury, (iii) epidemiology (age, gender, and career), (iv) classification/grade of injury, (v) symptoms, (vi) clinical examination, (vii) investigations, (viii) conservative management (indications and plan), (ix) operative management (indications, graft, and fixation types), (x) time to return to play, and (xi) post-operative complications.

## Results

**Figure 1:**
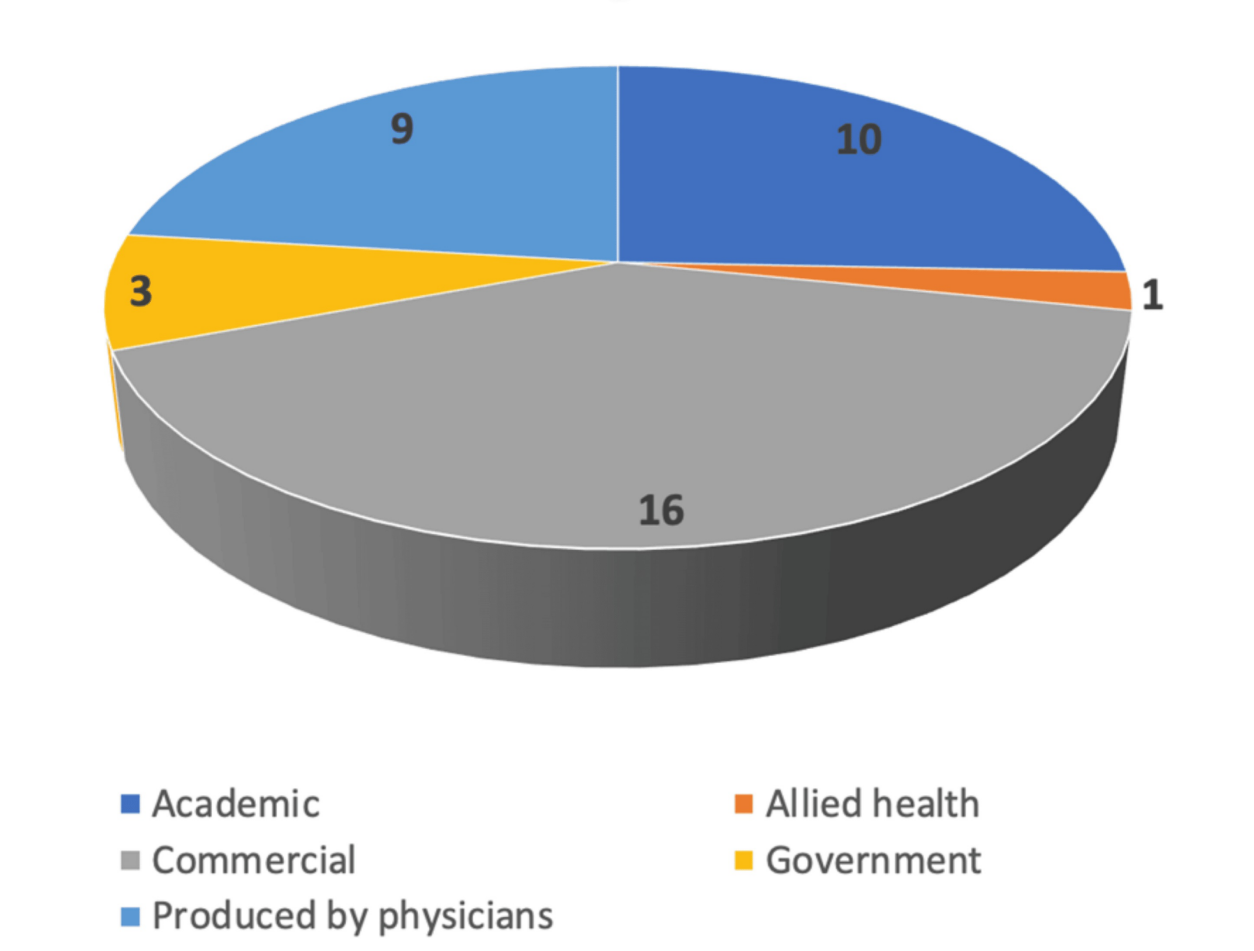
Nature of online websites

A total of 39 websites were included in this study. The nature of the online websites is summarised in Figure 1. The majority of websites were commercial (n = 16 [41.0%]), followed by academic (n = 10 [25.6%]). None of the websites included had a HON code present.

ACL content score

The mean ACL content score was 7.49 (SD = 2.04, range: 3-11). The mean highest score was achieved by websites produced by physicians, 8.11 (SD = 1.54), and government websites achieved the lowest, 6.33 (S.D = 1.53). There was no statistically significant difference between the ACL content scores across website nature (p=0.398).

JAMA score

The mean JAMA score was 2.62 (SD = 1.35; range: 0-4). The mean highest score was achieved by websites produced by physicians, 3.56 (SD = 0.527). Commercial (mean = 2.00, SD = 1.46) and government websites (mean = 2.00, SD = 2.00) achieved the lowest score. There was a statistically significant difference between the JAMA content scores across websites of different natures (p = 0.034).

DISCERN score

The mean DISCERN score was 52.1 (SD = 10.53; range: 32-70). The mean highest score was achieved by academic websites, 57.8 (SD = 11.2), and commercial websites achieved the lowest, 47.4 (SD = 9.30). There was no statistically significant difference between the DISCERN score across website nature (p = 0.180) (Table 1).

**Table 1:** Summary of results according to scoring systems

Type of website	No.	JAMA		ACL content score		DISCERN	
		Mean	SD	Mean	SD	Mean	SD
Overall	39	2.62	1.35	7.49	2.04	52.1	10.53
Academic	10	2.90	1.10	7.50	2.72	57.8	11.2
Allied health	1	-	-	-	-	-	-
Commercial	16	2.00	1.46	7.13	1.75	47.4	9.30
Government	3	2.00	2.00	6.33	1.53	49.0	7.21
Produced by physicians	9	3.56	0.527	8.11	1.54	53.3	9.22

## Discussion

Over the past decade, there has been a surge in the accessibility of online information with regard to healthcare [3]. In the United Kingdom, statistics showed that 61% of people between the ages of 16 and 24 used the internet to search for online health information [11]. One of the drawbacks of this behaviour is the potential for adverse effects, such as the development of anxiety [12]. Another potential harm is the possibility of attempting inappropriate treatment [13].

Patients are known to seek information through online resources prior to seeing a physician for various reasons [14]. It has also been demonstrated in previous similar studies that patients tend to seek information from the websites on the first page of search results [15]. Based on that, we chose the first 26 websites from Google and the first 13 websites from both Yahoo and Bing search engines. This was calculated based on search market engine shares of the respective search engines [7].

The results of this study showed that academic websites achieved the highest mean DISCERN score, while websites produced by physicians achieved the highest mean scores for the JAMA benchmark criteria and ACL content. This has been reflected in previous literature as well [16,17].

Devitt et al. have published a study relating to the information on the treatment of ACL injuries that included anterolateral ligament reconstruction (ALLR) [18]. This study has revealed findings that align with our own research, such as the correlation between DISCERN and their content-specific scores. However, this study only looked at information related to ALLR rather than ACL injuries as a whole, regardless of other treatment modalities.

HONcode-certified websites have been associated with higher DISCERN and JAMA scores. In addition, previous studies have demonstrated that HONcode-certified websites are linked with higher content-specific scores [19]. Our results noted that none of the websites were HONcode certified.

The following limitations of this study have been acknowledged by the authors: First, websites that required paid access or registration along with video-only explanatory content were excluded, potentially excluding well-established websites. Furthermore, the search was carried out using a particular search term of “anterior cruciate ligament injury,” and it should be noted that any other search term may have altered the websites that were produced by the search engines. We also assumed that the best resources available would be found on the most popular search engines: Google, Yahoo!, and Bing [7]. However, the popularity of search engines is something that is liable to change at a particular point in time. Finally, we also acknowledge that social media platforms are quite commonly used nowadays as a gateway to online information, but this is something that can be looked at in future studies.

## Conclusions

There is a vast amount of information available on the internet with regard to ACL injuries, and this can range from poor to excellent quality information. In light of this, it is essential that orthopaedic surgeons and various other healthcare providers of the multidisciplinary team guide patients to online resources that are reliable and reputable based on either their personal experience or resource type - in particular, physician and academic websites.
